# 

**Published:** 1896-02-22

**Authors:** 


CONTENTS.

Helpers or Rivals? The Nurse Question - - - - 339

The British Army as a Fighting Force - - - - 340

On Modern Progress in Ophthalmic Medicine and Surgery.—Latent Squint (Heterophoria). By R. B. CARTER, F.R.C.S. - - - - 341

Carbohydrate Starvation in Typhoid Fever. By ERIC PRITCHARD, M.A., M.B. - - - - 342

Annotations.—The Increase of English Suicides—A Danger to the Medical Profession—A Novel Cure for Colds - - - - 343

Notes and News - - - - 344

Editor's Letter Box - - - - 350

The Book World of Medicine and Science - - - - 353

Medical Progress and Hospital Clinics—

The Treatment of Fractures of the Patella. By J. POLAND, F.R.C.S. - - - - 345

PROGRESS IN MEDICINE.—On Diphtheritic Neuritis - - - - 347

THERAPEUTIC NOTES - - - - 349

PHYSICAL TREATMENT IN HEART DISEASE - - - - 349

THE CLINICAL SOCIETY - - - - 350

NEW APPLIANCES AND THINGS MEDICAL - - - - 350

The Institutional Workshop—

CONSTRUCTION NOTES.—The Caxton Convalescent Home, Limpsfield, Surrey - - - - 351

HOSPITAL MEETINGS - - - - 352

EXTRA SUPPLEMENT.—THE NURSING MIRROR.

News from the Nursing World.—The Patients' Treat—The Wreck of an Ambulance—Kent and Canterbury Nursing Institute—Private Nurses at Leeds—A Share in a Room—Queen's Nurses at Inverness—The Hospital Ship "Coromandel"—Nurses for Lepers—Hospital for Sick Children, Brighton—Enthusiastic but Portionless Workers—Hospital Sketches—Short Item - - - - clxxvii

Lectures on Nursing. X.—Diet, Recipes, Diabetes - - clxxix

Private Nursing. II.—Private Nurses Past and Present - - clxxx

Dress and Uniforms.—St. Bartholomew's Hospital - - clxxxi

St. Luke's Home, Vancouver - - - - clxxxii

Temperance for Nurses - - - - clxxxii

A New "Home for the Dying" - - - - clxxxii

A Book and Its Story - - - - clxxxiii

A Swiss Hospital - - - - clxxxiv

Practical Points - - - - clxxxiv

Everybody's Opinion - - - - clxxxv

Novelties for Nurses - - - - clxxxv

The Position of Nurses - - - - clxxxv

Appointments - - - - clxxxv

For Reading to the Sick - - - - clxxxvi

Notes and Queries - - - - clxxxvi

